# First determination of fullerenes in the Austrian market and environment: quantitative analysis and assessment

**DOI:** 10.1007/s11356-017-0213-x

**Published:** 2017-10-19

**Authors:** Susanna Zakaria, Eleonore Fröhlich, Günter Fauler, Anna Gries, Stefan Weiß, Sigrid Scharf

**Affiliations:** 1 Department of Medical Science, Medical University of Graz, Stiftingtalstrasse 24, 8010 Graz, Austria; 2 Department of Medical and Chemical Diagnostics, Medical University of Graz, Auenbruggerplatz 15, 8036 Graz, Austria; 3 Austrian Environment Agency, Spittelauer Lände 5, 1090 Vienna, Austria

**Keywords:** Fullerenes, Austrian environment, Wastewater, Sewage sludge, Cosmetics, LC-MS/MS, Carrez-clarification

## Abstract

This study forms the first report on analyzing fullerenes in the Austrian environment and cosmetic products available on the Austrian market. We developed, optimized, and validated a novel method for the analysis of C_60_ and C_70_ fullerenes and *N*-methylfulleropyrrolidine C_60_ (NMFP) for measuring sensitivities in the low nanograms per liter range in order to prove their presence in the environment (12 wastewater- and 12 sewage sludge samples) and in 11 selected fullerene-containing cosmetic products from three different brands. The optimized method relies on a liquid-liquid extraction (LLE) or solid-liquid extraction (SLE) and, for the first time, introduced the Carrez-clarification, followed by liquid chromatography (LC) and coupled to a hybrid triple quadrupole mass spectrometry (MS) quantification. The total variability of the new established LC-MS/MS method based on all the tested matrices was below 10%. We found recoveries generally higher than 70% for both tap water and surface water. The limits of quantitation (LOQ) for the wastewater samples were measured to be from 0.8 to 1.6 ng/L, for the sewage sludge samples, from 1.4 to 2.6 ng/g DM (drymass), and for the cosmetic samples from 0.2 to 0.4 ng/g. None of the analyzed samples of wastewater or sewage sludge samples contained fullerenes. But in 70% of the tested cosmetics, fullerene concentrations between 10 and 340 ng/g were detected. These values were much lower than concentrations causing toxicity in water animals.

## Introduction

Fullerenes were discovered 1985 by *Curl*, *Kroto*, and *Smalley* and belong to the class of carbon-containing nanomaterials (Thilgen 2001). Naturally, they occur in the form of various rocks, like in Shungite (Russia), in chalk-tertiary boundary layers (New Zealand), meteorites and meteoric impacts (Gissar, Tajikistan), and in strongly carbonaceous substrates, which are caused by lightning strike (Curl 1997). Fullerenes are able to form part of fumes generated by welding, metal smelting, jet engines, and automobile exhaust, where they are unintentionally released into the environment (Farre et al. 2010).

Due to their stability, size, hydrophobicity, three-dimensionality, electronic configurations, and wide range of possibilities of modification, they are attributed to a great use in biomedicine and material science to improve the material properties and product quality (Salomon 2006). For example, they can be used as radical scavengers in anti-aging creams, for the production of singlet oxygen in cancer therapy, for improving the material properties of solar cells, in sports articles (tennis, badminton, or golf), and for energy-saving and cost-effective conversion into diamonds, as opposed to graphite (Thilgen 2001).

By using fullerene-containing cosmetics, there is a direct contact and release into wastewater by rinsing the skin after the product use. Boxall et al. (2007) reported about 5–6% fullerene fractions in certain cosmetic products. Hansen et al. (2008) estimated the daily exposure to skin contact with a maximum of 26 μg/kg body weight. This corresponds to the intake of 0.1% fullerenes in cosmetic products. Due to the wide range of fullerene’s usage, nanomaterials are also becoming more and more involved in legislation, e.g., in the cosmetics-or biocide decree. Working groups at the European Chemicals Agency (ECHA) and Competent Authorities for REACH and CLP (CARACAL) are currently working on the improved integration of nanomaterials into the chemicals decree REACH. Since mid-2013, there has been a mandatory notification and labeling requirement for nanoparticles in cosmetics (EU Cosmetics Ordinance No. 1223/2009).

Already substantial measuring values of fullerene concentrations became certain in some studies of sewage sludge, waste water, and soil samples, as for example, Farre et al. (2010) and Emke et al. (2015) reported in Spain, Bruchet et al. (2013) in France, Carboni et al. (2016) in the Netherlands, and Sanchís et al. (2012) in the atmosphere of the Mediterranean Sea of Barcelona, Istanbul, and Alexandria. Due to the absence of standardized measuring methods in Austria, there are currently no data available for a quantitative exposure estimation.

In order to determine the quantity of fullerenes in the Austrian environment and a possible environmental risk assessment, previously mentioned analytical methods have to be optimized, since the analysis of new matrices, like hydrophobic cosmetics, require the development of suitable methods.

Additionally, there is barely any knowledge about the fate of fullerenes, if their concentration is increased in a certain environment. The exposure to a wide range of nanoparticles and the extent of a possible environmental damage and human burden are currently also not explained. Tiwari et al. (2014) suggested in their study that fullerenes may be degraded due to biotic and abiotic processes. On the other hand, Avanasi et al. (2014) and Navarro et al. (2017) described the stability of C_60_ that may result from their accumulation in the environment.

Due to these facts, the main motivation and objectives of the present study were to develop a suitable analytical method based on LC-MS/MS for quantifying fullerenes in wastewater, sewage sludge, and fullerene-containing cosmetic products. Especially C_60,_ C_70_ and NMFP fullerenes were chosen, because they are expected to be more abundant in the environment, due to the higher production volumes and their higher natural occurrence (Farre et al. 2010).

We described, for the first time in Austria, concentration levels of fullerenes in 24 wastewater- and sewage sludge samples, taken and analyzed in two rounds (September 2013 and December 2013), and also of 11 fullerene-contained cosmetic samples to assess the influence which these possible sources may have in the environmental occurrence.

In the future, these estimated levels could provide relevant data to evaluate the possible impact of carbon-based nanoparticles all over the world. Toxicity data obtained in studies on fullerene action in freshwater animals and plants are taken as indication of hazardous concentrations. According to toxicity tests by Zhu et al. (2006), 800 ng/g were determined as LD_50_ in *Daphnia magna*. Oberdörster showed as early as 2004 that 500 ng/g can cause oxidative stress in the brains of trout fish. Many studies also showed a cytotoxic effect of fullerenes in mouse models. For example, Tsuchiya et al. (1996) demonstrated a toxic effect on the embryos after intraperitoneal administration of 50 mg/kg of fullerenes into pregnant mice. Snyder et al. (2015) also reported that the fetuses died after 24 h, at most 8 days after injection, in pregnant and lactating rats exposed to ^14^C_60_ (0.2 mg/kg)-labeled fullerenes.

## Materials and methods

### Reagents and devices

Fullerene standards were purchased from Sigma-Aldrich (Steinheim, Germany), fullerene C_60_ (98% purity, CAS: 99685-96-8), [5, 6]-fullerene-C_70_ (98% purity, CAS: 115383-22-7), and *N*-methylfulleropyrrolidine C_60_ (99% purity, CAS: 151872-44-5). Twenty to thirty percent C-13 enriched 99+% C_60_ (CAS: 99685-96-8) were supplied from MER Corporation (Arizona, USA).

HPLC-grade methanol (MeOH) was supplied by Merck (Darmstadt Germany, CAS: 67-56-1), HPLC-grade toluene by PESTINORM® SUPRA TRACE by VWR Chemicals (Vienna Austria, CAS: 108-88-3), and HPLC-grade water by Promochem (Wesel Germany, CAS: 7732–18-5). Acetic acid 100% (HAc) was purchased by Merck (Darmstadt Germany, CAS:64-19-7), sodium chloride (NaCl) EMSURE® by Merck (Darmstadt Germany, CAS:7647-14-5), potassiumhexacyanoferrat (II)-trihydrate (Carrez-solution I) EMSURE® by Merck (Darmstadt Germany, CAS: 14459-95-1), and zinc sulfate-heptahydrate (Carrez-solution II) EMSURE® by Merck (Darmstadt Germany, CAS: 7446-20-0).

The stock solutions of fullerenes were prepared by adding 10 mg of the standards to 50-mL toluene with a final concentration of 0.2 mg/mL, sonication for 1 h in a water bath and storage in amber vials at 4 °C. Working solutions were prepared every week by appropriate dilution of the standard solution in toluene/methanol (10:90 *v*/*v*).

An ultrahigh-performance liquid chromatography (UPLC) system (Agilent Technologies 1290 Infinity Series) was used, equipped with a quaternary pump, autosampler, and column oven. The chromatographic separation was performed using a Luna C18 reversed-phase liquid chromatography column from Phenomenex (particle size 100 × 2 mm; 5 μm; 100 A) using toluene/methanol as mobile phase, gradient elution at a flow rate of 400 μL/min, and a column temperature of 30 °C.

The UPLC system was coupled to a triple-quadrupole mass spectrometer from Applied Biosystems-Sciex, API 4000 Q TRAP and equipped with an electrospray ionization Turbo Ion Spray source, working in negative electrospray ionization mode (ESI(-)).

Data analysis and evaluation were carried out with the AB Sciex Analyst Version 1.6.1 or MultiQuant 2.0 software.

### Sampling

Sampling was performed in cooperation with the NanoDESTINARA project 2015.

Wastewater and sewage sludge samples were collected in two rounds from five representative wastewater treatment plants (WWTP) from all over Austria. Twelve samples were collected in September 2013 and other 12 samples in December 2013. All samples were stored at − 20 °C in aluminum bottles (Table 1). Tap water was used as blank.

**Table 1:** List of 24 wastewater-and sewage sludge samples from 5 representative Austrian waste water treatment plants (WWTP)

Sample no.	Sample type	First round	Second round
1	WWTP 1 discharge	17.09.13	03.12.13
2	WWTP 2 discharge	17.09.13	03.12.13
3	WWTP 3 discharge	17.09.13	03.12.13
4	WWTP 4 discharge	17.09.13	03.12.13
5	WWTP 5 discharge , 1.stage	17.09.13	03.12.13
6	WWTP 5 discharge , 2.stage	17.09.13	03.12.13
7	WWTP 1 sewage sludge	17.09.13	03.12.13
8	WWTP 2 sewage sludge	17.09.13	03.12.13
9	WWTP 3 sewage sludge	17.09.13	03.12.13
10	WWTP 4 sewage sludge	17.09.13	03.12.13
11	WWTP 5 sewage sludge, 1.stage	17.09.13	03.12.13
12	WWTP 5 sewage sludge, 2.stage	17.09.13	03.12.13

The 11 cosmetic products from three different brands were selected after an extensive Internet- and literature research (Benn et al. 2011a) based on labeling, that the products contain “fullerenes.”

The cosmetic samples varied from medium to low water matrices: two were of medium- and nine of low water content. An antiaging cream (Diadermine, Austria) that did not comprise fullerenes was used as blank to represent a common cosmetic cream matrix.

### Sample treatment and extraction method

Fullerenes were extracted from homogenized wastewater, sewage sludge, and cosmetic samples by using liquid-liquid extraction ((LLE) Farre et al. 2010; Xia et al. 2006) or solid-liquid extraction (SLE). Both extraction methods are processed in the same manner; the only difference is that the liquid-liquid extraction is used exclusively for the wastewater samples or the high-water-content cosmetic samples. The solid-liquid extraction was used especially for the sewage sludge samples and cosmetic samples. In addition to the solid-liquid extraction, we used for the first time in the fullerene analytics a clean-up step called Carrez-clarification to remove interfering compounds from cosmetic samples with a high fat content, which can otherwise impair the LC-MS/MS analysis.

Cosmetic products with sample numbers 2, 3, 5, 7, and 9 were additionally purified with the Carrez-clarification.

Before starting the extraction, the isotope-labeled surrogate ^13^C_60_ was spiked to each sample and standard. The surrogate serves as relative reference (Huczko et al. 2000; Klaine et al. 2012), changing its concentration during the sample preparation. It is assumed that the concentration of the analyte changes in the same way. The surrogate was prepared with a final concentration of 1 ng/mL of ^13^C_60_ fullerene (c = 100 ng/mL in toluene) in 1-mL toluene. The dotation mix was prepared with a final concentration of 1 ng/mL of C_60_, C_70_, NMFP (c = 100 ng/mL in toluene) in 1-mL toluene.

LLE was carried out by taking 400 mL of homogenized wastewater samples and processed with 0.5-g sodium chloride and 40-mL toluene in a separating funnel. SLE was accomplished by taking 0.05–0.2 g of the cosmetic samples, 0.5–1.0-g sodium chloride, and 11.0-mL glacial acetic acid (100% GAA) in order to control the emulsion and addition of 5.0-mL toluene. The additional Carrez-clarification was performed in two steps by adding at first 1 mL of Carrez-solution I (150 g potassium hexacyanoferrat (II)-trihydrate in 1 L HPLC-water) and then another 1 ml of Carrez-solution II (300 g zinc sulfate in 1 L HPLC-water) for precipitation.

These mixtures were shaken for 1 h on the rotator and after phase separation, toluene was removed and centrifuged (Centrifuge Typ 3-18K Sigma) at 4000 rpm for 15 min. The supernatant was completely dried by evaporation (SuperVap™ Concentrator) using nitrogen in order to remove GAA, which could interfere with mass spectrometric detection. The other method without Carrez-clarification included evaporation of the supernatant to a volume of 0.1 mL and adjustment to 1.0 mL with methanol in an amber-glass injection vial for quantification by LCMS.

### Analysis by LC-MS

C_60_, C_70_, and NMFP were quantified in the toluene extracts with methanol using LC-MS with ESI in negative mode. Chromatographic separation was achieved with a Luna 5u C18 (2), 100A (100 × 2.0 mm) column with a constant flow rate of 400 μL/min. The analytes were eluted with a gradient, using toluene and methanol.

Substance-specific MS parameters were first optimized by infusion, followed by the chromatographic conditions and the source-dependent MS parameters through flow injection analysis (FIA).

The process control and the reliable reproducibility of the method were ensured by using an isotope-labeled surrogate ^13^C_60_ fullerene (Bobylëv et al. 2012). The quality assurance was given by determining the linear range, the limit of detection (LOD), and the limit of quantitation (LOQ). The calculation of the LOD and LOQ was done by the basic validation according to DIN 32645 (Kolb et al. 2006). Therefore, the instrumental LOD and LOQ were determined by means of the validation software (SQS 2010 version 1.46) using the peak areas of at least three series of the standards. The five smallest concentrations had to be linear. The calculation was automatically done and defined by the software from the measurements. For the evaluation of the samples, the determined LOQ and LOD (LOQ/2) were derived from the calculated LOQ and LOD. LOQ was determined as the minimum detectable amount of analyte with a signal-to-noise ratio of 3:1, respectively. Linearity was based on the calibration curve of 9 standard points and was generated by means of quadratic regression and weighted to 1/× over the concentration range of 0.01–2.5 ng/mL. Standards above the 2.5 ng/mL were assayed, but not used for the calibration since the calibration curve was flattened and had a poorer signal intensity.

The analysis and evaluation of the mentioned fullerenes were carried out with the Analyst-Software 1.6 (Applied Biosystems-Sciex). The resulting chromatograms from the measurements were integrated and the concentrations were quantified using MultiQuant 2.0 software. All calculations were performed in Microsoft Excel 2010, based on data from the analyst software.

## Results and discussion

### Mass spectrometry

#### Substance-dependent MS-parameters

To determine the precursor- and product ions of the analytes, an infusion with a syringe pump in the tuning mode of the MS software was done. Therefore, 100 ng/mL of fullerene standard solutions were prepared in toluene/methanol (10:90 *v*/*v*) and infused at a flow rate of 20 μL/min using a syringe pump (Harvard Apparatus 11).

Molecular ions [M·−] were formed under ESI-MS conditions and were the most abundant ions including *m*/*z* 720 for C_60_, *m*/*z* 840 for C_70_, and *m*/*z* 777 for NMFP. Due to the chemical stability of fullerenes, C_60_ and C_70_ ions could not be fragmented_,_ because the precursor ions (Q1) also serve as product ions (Q3) (quasi-MRM transitions). We achieved the same results as previously published by Isaacson et al. (2007) and Farre et al. (2010).

In addition, the substance-dependent MS parameters (declustering potential (DP), entrance potential (EP), collision energy (CE), collision cell exit potential (CXP)) were optimized by gradual increase or decrease of each parameter to reach the maximum signal intensity for each ion (Table 2).

**Table 2:** Optimized substance dependent MS-Parameters in MRM Modus. Retention time (RT) in Minutes, transitions (Precursor und fragment-Ions (m/z)), Declustering Potential (DP) in Volt, Entrance Potential (EP) in Volt, Collision Energy (CE) in Volt, Collision Cell Exit Potential (CXP) in Volt

Fullerenes	RT [min]	Precursor-Ion [m/z]	Product-Ion [m/z]	DP [Volt]	EP [Volt]	CE [Volt]	CXP [Volt]
Fullerene C_60_	2.8	720.6	720.6	-120	-10	-5	-9
Fullerene C_70_	3,0	840.1	840.1	-120	-10	-5	-9
NMFP	2.41	777.1	720.6	-120	-10	-125	-25
Surrogat (^13^C_60_)	2.79	738.0	738.0	-120	-10	-5	-9

#### Source-dependent MS parameters

The optimization of the source-dependent MS parameters (curtain gas flow, ion source gas 1 (nebulizer gas) and ion source gas 2 (heating gas), Ion Spray transfer voltage, vaporizer temperature, and collision gas) was performed by using Flow Injection Analysis (FIA). Therefore, mixed standards of 1 ng/mL were prepared and injected into the mass spectrometer by means of several injections of the analyte and various variants of all the source-dependent parameters in order to determine the most optimal conditions (Table 3).

**Table 3:** Optimized source-dependent MS-Parameters

Source dependent-MS Parameters	unit
Curtain gas flow (CUR)	25
Ion source gas 1(GS1)	50
Ion source gas 2 (GS2)	50
Ion Spray transfer voltage (IS)	4200
Interface heater	ON
Vaporizer temperature [°C]	700
Collision Gas (CAD)	low

### Chromatography

#### Optimization of chromatographic separation

For the optimization of the chromatographic separation, we searched for the most ideal combination of the separation column and the eluent composition. According to Farre et al. (2010), C18 columns are used more often than Lichrolut ENV+, Oasis MCX, and Strata-X for the extraction of target compounds in environmental analysis, because they generally do not need pH adjustments or any special elution conditions. They also declare in their work that using columns with a particle diameter below 2 mm results in a better chromatographic resolution and increased peak capacity. Therefore, in this present work, a Luna 5u C18 (2), 100A (100 × 2.0 mm) column was used.

**Fig. 1:**
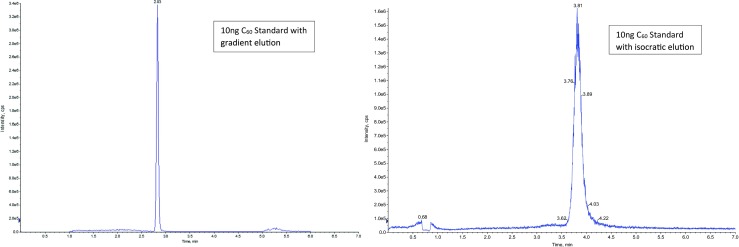
Ten nanograms C_60_ standard with gradient elution (left) and isocratic elution (right) are shown. Chromatograms are carried out with AB Sciex Analyst Version 1.6.1

Toluene was chosen as solvent according to Korobov and Smith (2000). They discovered that toluene is a better eluent compared to benzene or water with a solubility of 3.89 mmol/L for C_60_ and 1.67 mmol/L for C_70_. The tested substances could also be eluted almost completely by toluene. Toluene and methanol (LC/MS grade) served as eluents for the mobile phase. In order to improve the baseline separation as well as the signal intensity, multiple injections of different fluid composition were carried out in order to find the best combination. For the optimization of the gradient, a series of experiments with different compositions of the eluent and column oven temperature was essayed and the best one is shown in Table 4. Many studies, like Farre et al. (2010), Núñez et al. (2012), and Chao et al. (2011), used an isocratic elution for their analyzes. The use of a gradient comparatively resulted in a better signal intensity in this study because shorter retention time and a better peak shape were achieved (Fig. 1).

**Table 4:** Optimal gradient composition (A:Toluene B:MeOH)

Total Time (min)	Flow Rate (μl/min)	A (%) (Toluene)	B (%) (MeOH)	Temperature (°C)
0.00	400	40	60	30°C
1.00	400	40	60	30°C
2.00	400	70	30	30°C
4.00	400	70	30	30°C
4.10	400	40	60	30°C
7.00	400	40	60	30°C

### Recoveries of C_60,_ C_70_, and NMFP in tap water and surface water

Recovery experiments were performed to evaluate the efficiency of the liquid-liquid and solid-liquid method for extracting C_60_, C_70_, and NMFP. Benn et al. (2011a) evaluated three extraction methods, namely SPE, LLE, and sonication in toluene. LLE yielded the best recovery results (96 and 107%). SPE and sonication in toluene achieved much lower fullerene recovery efficiencies than LLE, ranging from 27 to 42%. This study also showed that the LLE extraction method was the most efficient one. Moreover, the liquid-liquid extraction was advantageous because highly particle-containing wastewater effluents could be extracted without previous filtration and possible target substances adsorbed to the particles could also be easily dissolved. Furthermore, investigations of the NanoDESTINARA project 2015 showed that fullerenes have the tendency to adhere to solids. In their adsorption tests, over 90% of fullerenes were found in sewage sludge (Kreuzinger et al. 2016). These results also lead to the conclusion that the LLE method is appropriate for fullerene analysis.

The first liquid-liquid extraction of C_60_, C_70_, and NMFP was carried out with tap water and surface water (Danube 2013) to minimize the possible initial presence of fullerenes. The recovery experiments were done with 400 mL of each type of water and fortified with a dotation mix and ^13^C_60_ surrogate mix in the same concentration as described above in 2.3.

Results of the mean values (MV) and standard deviations (STADV) of the recoveries (RV) in the tap water and surface water from three experiments are listed in Table 5. Kolkman et al. (2013) described the recoveries in water in a similar order of magnitude. The recoveries in the undiluted samples are lower, indicating matrix effects, which could be reduced by dilution.

**Table 5:** MV und STADV of undiluted and (1:10) diluted recoveries (RV in %) in tap water and surface water

Matrix	MV und STADV (%)		MV und STADV (%)		MV und STADV (%)	
Tap water	C_60_		C_70_		NMFP	
RV	61.9	5.1	57.1	4.7	54.6	8.9
RV (1:10)	86.7	6.4	81.8	8.2	75.7	8.6
Surface water	C_60_		C_70_		NMFP	
RV	55.0	3.5	44.9	4.5	47.1	8.7
RV (1:10)	76.5	6.0	48.2	5.3	71.7	8.0

LOD for C_60_ and NMFP were 0.30 ng/L, for C_70_ 0.6 ng/L. LOQ for C_60_ and NMFP were 0.60 ng/L, for C_70_ 1.20 ng/L. Compared to Kolkman et al. (2013), the LOD and LOQ for C_60_ are approximately in the same order of magnitude as described in the present work (LOD 0.28 ng/L and LOQ 0.57 ng/L).

### Results of the wastewater and sewage sludge samples

**Fig. 2:**
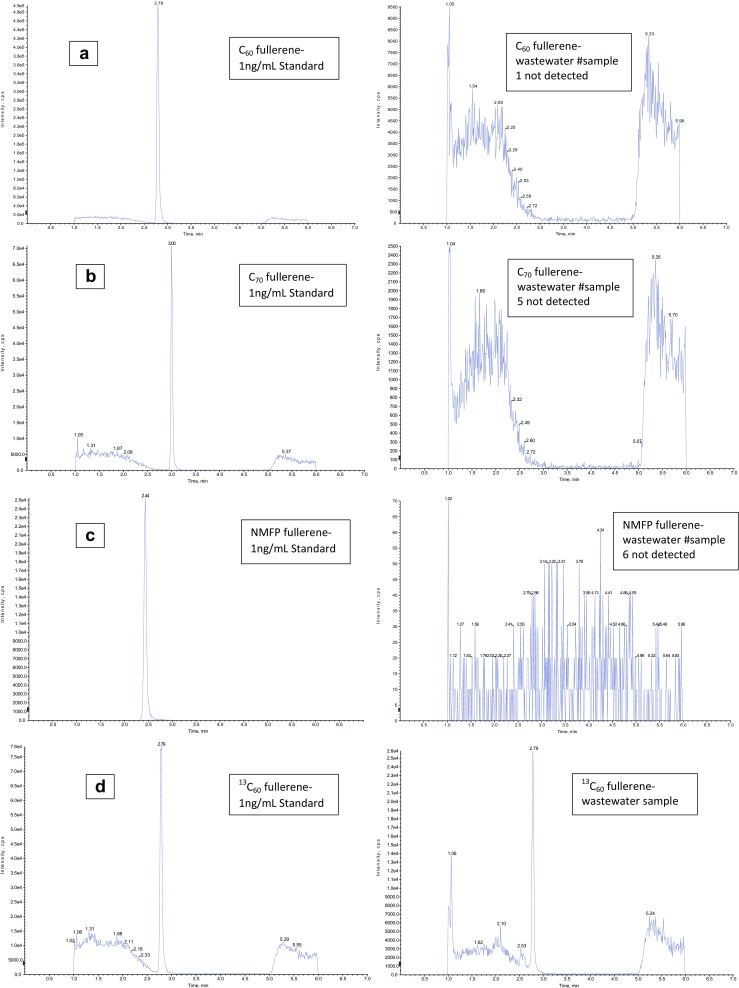
Chromatograms **a** #sample 1, **b** #sample 5, **c** #sample 6, and **d** ^13^C_60_ internal standard are carried out with AB Sciex Analyst Version 1.6.1. One nanogram per milliliter standards (left) and wastewater samples (right) are shown. Elution time is plotted on the *x*-axis up to 7 min. The *y*-axis shows the maximum intensity of each fullerene in cps

**Fig. 3:**
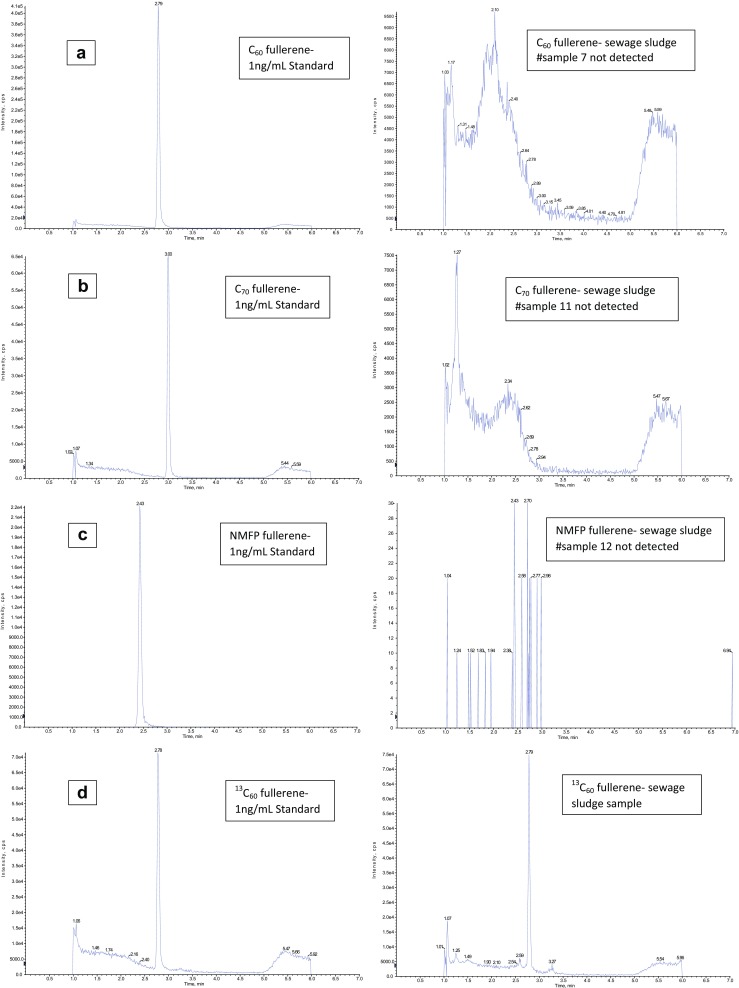
Chromatograms **a** #sample 7, **b** #sample 11, **c** #sample 12, and **d** ^13^C_60_ internal standard are carried out with AB Sciex Analyst Version 1.6.1. One nanogram per milliliter standards (left) and sewage sludge samples (right) are shown. Elution time is plotted on the *x*-axis up to 7 min. The *y*-axis shows the maximum intensity of each fullerene in cps

Some representative chromatograms of the 12 waste water and 12 sewage sludge samples are shown in Figs. 2 and 3.

With this method, a LOQ of 0.80 ng/L and LOD of 0.40 ng/L was achieved for C_60_ and NMFP, and a LOQ of 1.60 ng/L and LOD of 0.80 ng/L was achieved for C_70_. Surrogate recoveries in wastewater samples were found in the range of 60–80%. In the 12 wastewater samples, which were analyzed in two periods, no fullerenes were detected above the LOD. Farre et al. (2010) reported a LOQ ranging from 0.2 to 1 ng/L for wastewater.

Futhermore, a LOQ of 1.4 ng/g dry mass (DM) and LOD of 0.7 ng/g DM was achieved for C_60_ and NMFP and a LOQ of 2.6 ng/g DM and LOD of 1.3 ng/g DM for C_70_. The dry mass of the sewage sludge samples was between 0.2 and 0.5% (median: 0.35%). In the 12 sewage sludge samples which were also analyzed in two periods, no fullerenes were detected above the LOD.

Kreuzinger et al. (2016) come to the conclusion in their work “Synthetic nanoparticles in wastewater treatment” that the flow inlet also does not contain any fullerenes, which is comparable to our results.

### Optimization of the solid-liquid extraction

**Fig. 4:**
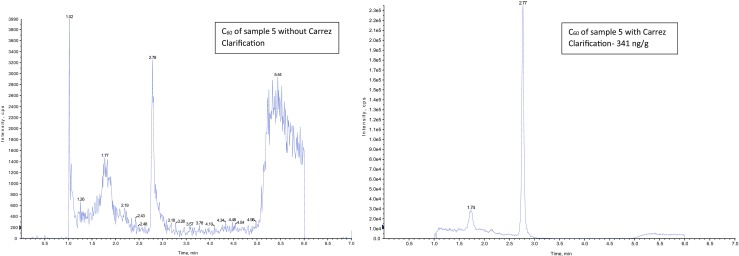
Sample 5 without Carrez-Clarification (left) and with Carrez-Clarification (right). Chromatograms are carried out with AB Sciex Analyst Version 1.6.1

The usage of solid-liquid extraction in hydrophobic cosmetics results in lower peak sensitivities, due to very high fat levels in the products. Therefore, we optimized the SLE method by additional use of the Carrez-clarification. Figure 4 shows significantly better results, by comparing the samples with or without the extra clean-up step. Therefore, we can show a clear improvement in sample purity by using Carrez-clarification especially for hydrophobic cosmetics, which not only shows better ability for quantitation of samples but also leads to longer life time of chromatographic columns and better performance of the analytic instrument.

### Results of the cosmetic samples

**Fig. 5:**
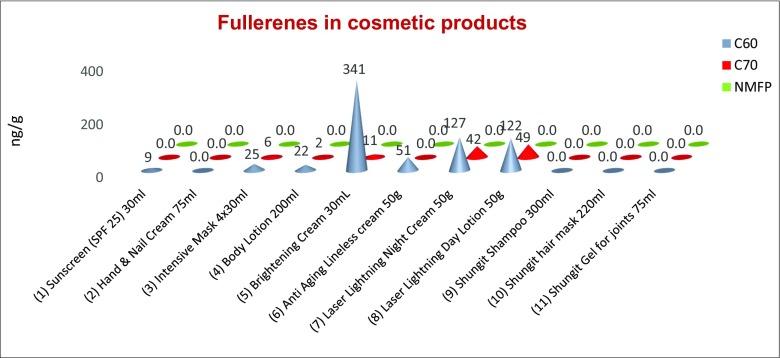
Results of the 11 cosmetic products are carried out with Excel 2016. C_60_ values are shown in blue (first line), C_70_ values in red (second line), and NMFP values in green (third line). All the results are indicated in nanograms per gram

With the optimized method concentrations above the LOD for C_60_ and C_70_ ranging from 10 to 340 ng/g in 8 of the 11 samples were detected, no fullerenes were detectable in 3 of the cosmetic products (Fig. 5). NMFP was not detected in all the analyzed products. Benn et al. (2011b) also found fullerenes in a similar range for example C_60_ 0.04–1.1 μg/g and for C_70_ 0.07 μg/g. With this method, a LOQ of 0.2 ng/g and LOD of 0.1 ng/g was obtained for C_60_ and NMFP, and a LOQ of 0.4 ng/g and LOD of 0.2 ng/g was obtained for C_70_. Benn et al. (2011b) calculated a LOQ of 12.0 ng/g and LOD of 3.0 ng/g for C_60_ and C_70_. Compared with the present literature, the detection limit for the tested substances could be undercut.

We found out that the use of fullerene-containing cosmetic products like C_60_ or C_70_ represents an entry point into the environment.

## Conclusion

The analysis of C_60_, C_70_, and NMFP fullerenes in wastewater- and sewage sludge samples were carried out with the optimized chromatographic conditions of the established method. The method was characterized by determining the linear range, the LOQ, and the LOD. Notable in this work is the usage of an additional clean-up step, namely the Carrez-clarification and the application of the gradient elution which proved to be a suitable optimization for the analysis of fullerenes in environmental samples and cosmetic products. The results showed that C_60_, C_70_, and NMFP fullerenes could not be found above the limit of detection in the tested samples of WWTP. We assume that considerable fullerene concentrations in wastewater treatment plants of other European countries like Spain, the Netherlands, or France are due to the higher consumption of fullerenes in those countries. Possibly, they also can be explained by the purification quality of the wastewater treatment plants itself.

Fullerenes in cosmetics were measured partially in high nanograms per milliliter and nanograms per gram concentrations, whereas some branded products, such as EVIDENS de Beaute and Dr. Brandt. Shungit products showed no detectable fullerene concentrations.

The results of the present study suggest that the Austrian population already comes into contact with products containing fullerenes, but there is still a lack of knowledge about the fate and transport of fullerenes to definitely exclude a danger for the environment or human health.

However, this could change with the increasing trend and consumption of products containing fullerenes over the coming next years. Therefore, continuous monitoring is necessary to ensure the safety of the population and the environment. Furthermore, there should be investigations to standardize materials and test methods with reference particles, so that future data can be assuredly compared with each other.
